# Isolated recurrence of distal adenocarcinoma of the extrahepatic bile duct on a draining sinus scar after curative resection: Case Report and review of the literature

**DOI:** 10.1186/1477-7819-7-96

**Published:** 2009-12-14

**Authors:** Jesús Rodríguez-Pascual, Emilio De Vicente, Yolanda Quijano, Francisco Pérez-Rodríguez, Fernando Bergaz, Manuel Hidalgo, Ignacio Duran

**Affiliations:** 1 Centro Integral Oncológico Clara Campal (CIOCC), Madrid, Spain; 2 Surgery Department, Hospital Madrid-Norte Sanchinarro, Madrid, Spain; 3 Pathology Department, Hospital Monteprincipe, Boadilla del Monte, Madrid, Spain; 4 Radiology Department, Hospital Monteprincipe, Boadilla del Monte, Madrid, Spain

## Abstract

**Background:**

Surgical resection remains the gold standard for the treatment of localized adenocarcinoma of the extrahepatic bile ducts. Yet, treatment of loco-regional recurrences is not well defined.

**Case Presentation:**

We present an unusual case of distal adenocarcinoma of the extrahepatic bile ducts that was treated with surgery and relapsed two years later with a solitary recurrence on the tract of a previous Redon drain. In addition, a review of the literature on management of loco regional relapses is presented.

**Conclusions:**

The ideal management of these patients still remains undefined. Decisions are made based on clinical parameters from retrospective series, such as tumor grade, surgical margins or lymph node involvement. Prospective studies, that include molecular and genetic markers, are needed to improve patient selection and outcomes on this population.

## Background

Biliary tract neoplasms are relatively infrequent malignancies. Adenocarcinoma of the extrahepatic bile duct comprises around half of all bile duct neoplasms. Surgical resection remains the gold standard for this disease although adjuvant chemotherapy or radiation should be considered in selected cases. Treatment protocols for loco-regional recurrences are not well defined and include surgery, chemotherapy, radiation, or best supportive care. We present an unusual case of adenocarcinoma of the extrahepatic bile duct treated with surgery that relapsed two years later with a solitary recurrence on the tract of previous Redon drain.

## Case presentation

**Figure 1:**
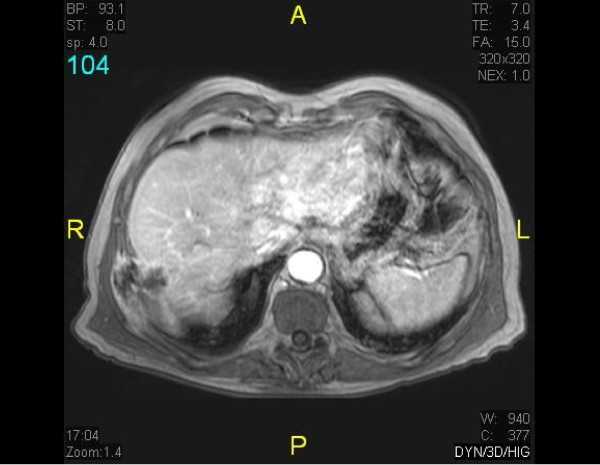
**Abdominal MRi**. Isolated recurrence in chest wall of a previously resected cholangiocarcinoma: long string through the diaphragm, chest, and abdominal wall that extended from the surgical bed.

**Figure 2:**
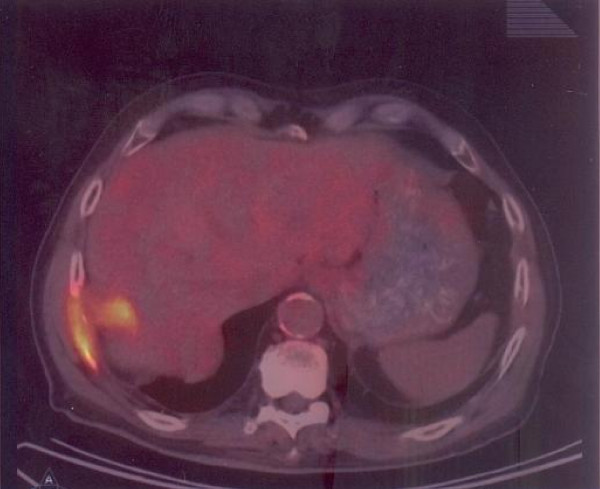
**PET-CT**. Isolated recurrence in chest wall of a previously resected cholangiocarcinoma: intense glucose uptake.

A 62 year-old Caucasian male presented with a seven-day history of painless jaundice with no other accompanying symptoms. His past medical history was remarkable for high blood pressure, hypercholesterolemia, coronary artery disease, tuberculosis, abdominal aortic aneurysm and benign essential tremor. At admission, physical examination revealed unremarkable findings aside from jaundice. Imaging tests showed a hilar-hepatobilliary mass consistent with the diagnosis of adenocarcinoma of the extrahepatic bile ducts (extrahepatic cholangiocarcinoma). On February 2003, the patient underwent a percutaneous biliary internal decompression followed by a laparotomy and curative resection of the tumour by cephalic duodenopancreatectomy. The resection was performed successfully and histological examination revealed an adenocarcinoma of the extrahepatic bile duct with no lymph node involvement and clear microscopic margins (pT2 pN0 as per AJCC classification)[1]. The patient did not receive any adjuvant therapy and his follow up consisted on CT scans, blood work, and physical exams every six months. Two years after the initial surgery, the patient complained of a four-month insidious and progressive abdominal pain localized in the right upper abdominal area along with a weight loss of around eight kilograms. Physical exam was unremarkable and abdominal imaging studies (including ultrasound and CT scan) reported only post-surgical changes with no evidence of recurrence. Blood work was notable for an elevated CA 19-9 at 880 Ku/L (normal range 0-39) with no other abnormalities. A liver MRI and PET-CT revealed a long string through the diaphragm, chest, and abdominal wall that extended from the surgical bed, with an intense glucose uptake at a standard uptake value of 7 (Figs 1 and 2). With the diagnosis of isolated recurrence in the chest wall of a previously resected cholangiocarcinoma, the patient underwent a new surgical intervention consisting of an en-bloc resection of segment VII of the liver, part of the diaphragm and the whole 11^th ^right rib.

**Figure 3:**
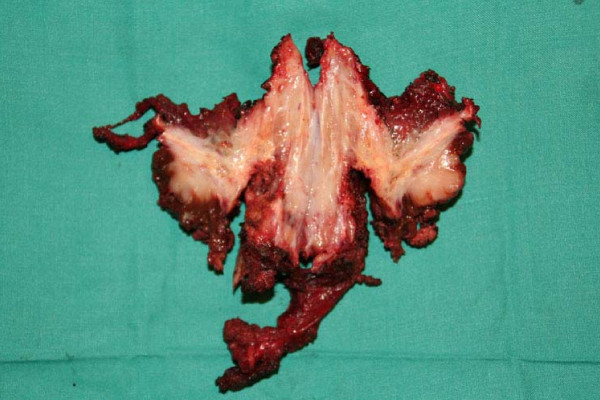
**Surgical specimen**. Tumour implant through the drainage course where the Redon tube was placed in the first liver procedure.

Pathology analysis revealed a tumour implant through the drainage course where the Redon tube was placed in the first liver procedure (Fig 3). No tumour was observed in the liver parenchyma and surgical margins were free of malignant cells.

After the successful resection of this unusual recurrence, the patient received six courses of adjuvant chemotherapy (gemcitabine and oxaliplatin) with good tolerance[2]. On December 2007, twelve months after finishing adjuvant treatment, the patient presented again with abdominal pain, fever, and hypotension. A mass in the right upper quadrant was palpated on physical exam and imaging studies revealed the presence of a 10 cm lesion involving the chest wall, liver, and lung. Puncture of the mass confirmed the presence of adenocarcinoma as well as a purulent collection complicating this lesion. In spite of multi-antibiotic treatment and drainage of the collection, the patient died on January 10^th ^2008 due to refractory septic shock.

## Discussion

### Management of resectable bile duct adenocarcinoma

Adenocarcinoma of the extrahepatic bile duct are relatively infrequent malignancies representing less than 5% of all solid tumors in adults and accounting for approximately 0.5% of all cancer deaths[3]. Adenocarcinomas of the extrahepatic bile duct (previously referred as extrahepatic cholangiocarcinomas) are also distinguished as proximal or distal, depending on their location along the bile ducts.

Surgery provides the only possibility for cure in these neoplasms. Distal cholangiocarcinomas have the highest resectability rates, while proximal tumors have the lowest (particularly perihiliar neoplasms) [4-6]. In one large series, the resectability rates for distal, intrahepatic and perihiliar lesions were 91, 60 and 56 percent respectively[7]. Even in patients who undergo potentially curative resection, tumor free margins can be obtained in only 20 to 40 percent of proximal and 50 percent of distal tumors[8]. These numbers are even lower if a proximal tumor free margin of at least 5 mm is considered to constitute a curative procedure[9]. Thus, although surgical resection remains the gold standard for this disease, long term survival is rarely achieved because of frequent postoperative recurrences [10,11].

The main clinical criteria for resectability are: absence of retropancreatic and paraceliac nodal metastases, no distance liver metastases or disseminated disease, absence of invasion of the portal vein or main hepatic artery (although in many cancer centers, including our institution, en bloc resection with vascular reconstruction can be considered), and absence of extrahepatic adjacent organ invasion [12].

Patients with positive margins after resection or positive regional lymph nodes should be considered for adjuvant 5FU-based chemotherapy as well as radiation. Yet, no randomized trials have been conducted that support a standard regimen. Individuals with negative margins after surgery and negative regional lymph node involvement can either be observed or treated with adjuvant strategies [13].

### Follow-up after resection and diagnosis of loco-regional relapse

No clear guidelines exist for follow-up after surgery in this particular tumor type. Physical exam with routine laboratory tests every 3 to 4 months for the first 3 years post-surgery and then at longer intervals of 6 months until year 5 seems a reasonable approach. The role of CA 19-9 level in surveillance is not clear, but persistently rising levels often precede radiological evidence of recurrence by a number of months. Therefore this marker has been routinely incorporated in follow-up schemas. Which imaging tests to be performed is a topic that has not been specifically addressed in prospective trials, although CT scans of the abdomen every 6 months for 2 to 3 years after surgery is probably the most common approach in routine practice. However, as referred in the case presented, in several occasions CT and abdominal ultrasound are not sufficient to detect loco-regional relapses, which could be easily determined on MRI and PET.

While recurrence is mostly loco-regional in the majority of proximal tumors, distal cholangiocarcinomas recur frequently at distant sites including the liver, peritoneum, and lung[14,15]. Like pancreatic, gallbladder and hepatocelular cancers, adenocarcinomas of the bile duct have a predisposition to seed an can recur in needle biopsy tracts, abdominal wall incisions wounds and the peritoneal cavity, and therefore it is recommended to be especially careful in the physical exams in each follow-up visit.

### Clinical management of loco-regional relapse

The case presented illustrates an example of an unusual pattern of relapse for adenocarcinoma of the extrahepatic bile duct on a draining sinus from a previous surgical procedure. Moreover it shows how the ideal management of these patients still remains undefined. No prospective data exist to set definitive recommendations about the optimum treatment after a curative resection of adenocarcinoma of the extrahepatic bile ducts. Currently, decisions are made based on different clinical parameters that have been established as prognostic factors in retrospective series, such as tumor grade, surgical margins, or lymph node involvement.

Surgery is generally not indicated for recurrent bile duct adenocarcinoma due largely to the location of recurrence, technical difficulty, frequent distant metastases and aggressiveness. However, in patients with prolonged relapse-free interval and favorable location, should be an option to consider.

More recently, several molecular markers have been explored as possible determinants of invasiveness and relapse. Expression of EGFR, HER2 and VEGF has been correlated with disease recurrence and could be incorporated into the decision-making process of deciding adjuvant treatment in this patient population [16]. Another retrospective analysis investigated the correlation of c-met, cox2, and IL6 expression with invasiveness and lymph node metastasis in a series of 114 patients [17]. Additionally, the importance of epigenetic alterations in the process of cholangiocarcinogenesis has been highlighted recently with respect to their potential as diagnostic and prognostic tools [18].

## Conclusions

The current management of patients with relapsed adenocarcinoma of the extrahepatic bile duct after resection of the primary tumor remains poorly defined and is based only on small retrospective series. Further prospective studies are required in order to design the most appropriate treatment strategy on this population. In addition to the clinical knowledge, it may be beneficial to incorporate molecular and genetic markers into the treatment decision algorithm of this neoplasm.

## Consent

Written informed consent was obtained from the patient for publication of this case report and any accompanying images. A copy of the written consent is available for review by the Editor-in-Chief of this journal.

## Competing interests

The authors declare that they have no competing interests.

## Authors' contributions

JRP and ID conceived the idea for the manuscript, conducted a literature search, and drafted the manuscript. EDV and YQ performed surgery, obtained specimen images and critically revised the manuscript. FPR provided and reviewed pathological images FB obtained radiological images used in the manuscript. MH critically revised the manuscript. All authors read and approved the final manuscript.

## References

[B1] Sobin LH Wittekind C TNM Classification of Malignant Tumours (UICC) 2002 6 New York: Wiley-Liss

[B2] Eckel F Schmid RM Chemotherapy in advanced biliary tract carcinoma: a pooled analysis of clinical trials Br J Cancer 2007 96 896 902 10.1038/sj.bjc.6603648 17325704 PMC2360111

[B3] Jemal A Siegel R Ward E Hao Y Xu J Murray T Thun MJ Cancer statistics, 2008 CA Cancer J Clin 2008 58 71 96 10.3322/CA.2007.0010 18287387

[B4] Nakeeb A Lipsett PA Lillemoe KD Fox-Talbot MK Coleman J Cameron JL Pitt HA Biliary carcinoembryonic antigen levels are a marker for cholangiocarcinoma Am J Surg 1996 171 147 152 discussion 152-143 10.1016/S0002-9610(99)80090-7 8554130

[B5] Tompkins RK Saunders K Roslyn JJ Longmire WP Jr Changing patterns in diagnosis and management of bile duct cancer Ann Surg 1990 211 614 620 discussion 620-611 1692678 PMC1358236

[B6] Schoenthaler R Phillips TL Castro J Efird JT Better A Way LW Carcinoma of the extrahepatic bile ducts. The University of California at San Francisco experience Ann Surg 1994 219 267 274 10.1097/00000658-199403000-00006 8147607 PMC1243134

[B7] Nakeeb A Pitt HA Sohn TA Coleman J Abrams RA Piantadosi S Hruban RH Lillemoe KD Yeo CJ Cameron JL Cholangiocarcinoma. A spectrum of intrahepatic, perihilar, and distal tumors Ann Surg 1996 224 463 473 discussion 473-465 10.1097/00000658-199610000-00005 8857851 PMC1235406

[B8] Burke EC Jarnagin WR Hochwald SN Pisters PW Fong Y Blumgart LH Hilar Cholangiocarcinoma: patterns of spread, the importance of hepatic resection for curative operation, and a presurgical clinical staging system Ann Surg 1998 228 385 394 10.1097/00000658-199809000-00011 9742921 PMC1191497

[B9] Sakamoto E Nimura Y Hayakawa N Kamiya J Kondo S Nagino M Kanai M Miyachi M Uesaka K The pattern of infiltration at the proximal border of hilar bile duct carcinoma: a histologic analysis of 62 resected cases Ann Surg 1998 227 405 411 10.1097/00000658-199803000-00013 9527064 PMC1191279

[B10] Kurosaki I Hatakeyama K Tsukada K Long-term survival of patients with biliary tract cancers with lymph node involvement J Hepatobiliary Pancreat Surg 1999 6 399 404 10.1007/s005340050139 10664290

[B11] Todoroki T Kawamoto T Koike N Takahashi H Yoshida S Kashiwagi H Takada Y Otsuka M Fukao K Radical resection of hilar bile duct carcinoma and predictors of survival Br J Surg 2000 87 306 313 10.1046/j.1365-2168.2000.01343.x 10718799

[B12] Chamberlain RS Blumgart LH Hilar cholangiocarcinoma: a review and commentary Ann Surg Oncol 2000 7 55 66 10.1007/s10434-000-0055-4 10674450

[B13] McMasters KM Tuttle TM Leach SD Rich T Cleary KR Evans DB Curley SA Neoadjuvant chemoradiation for extrahepatic cholangiocarcinoma Am J Surg 1997 174 605 608 discussion 608-609 10.1016/S0002-9610(97)00203-1 9409582

[B14] Jarnagin WR Ruo L Little SA Klimstra D D'Angelica M DeMatteo RP Wagman R Blumgart LH Fong Y Patterns of initial disease recurrence after resection of gallbladder carcinoma and hilar cholangiocarcinoma: implications for adjuvant therapeutic strategies Cancer 2003 98 1689 1700 10.1002/cncr.11699 14534886

[B15] Kitagawa Y Nagino M Kamiya J Uesaka K Sano T Yamamoto H Hayakawa N Nimura Y Lymph node metastasis from hilar cholangiocarcinoma: audit of 110 patients who underwent regional and paraaortic node dissection Ann Surg 2001 233 385 392 10.1097/00000658-200103000-00013 11224627 PMC1421255

[B16] Yoshikawa D Ojima H Iwasaki M Hiraoka N Kosuge T Kasai S Hirohashi S Shibata T Clinicopathological and prognostic significance of EGFR, VEGF, and HER2 expression in cholangiocarcinoma Br J Cancer 2008 98 418 425 10.1038/sj.bjc.6604129 18087285 PMC2361442

[B17] Joo HH Song EY Jin SH Oh SH Choi YK [Expressions and clinical significances of c-met, c-erbB-2, COX-2, and IL-6 in the biliary tract cancers] Korean J Gastroenterol 2007 50 370 378 18159174

[B18] Sandhu DS Shire AM Roberts LR Epigenetic DNA hypermethylation in cholangiocarcinoma: potential roles in pathogenesis, diagnosis and identification of treatment targets Liver Int 2008 28 12 27 18031477 10.1111/j.1478-3231.2007.01624.x PMC2904912

